# Exploring Prediction of Antimicrobial Resistance Based on Protein Solvent Accessibility Variation

**DOI:** 10.3389/fgene.2021.564186

**Published:** 2021-01-22

**Authors:** Simone Marini, Marco Oliva, Ilya B. Slizovskiy, Noelle Robertson Noyes, Christina Boucher, Mattia Prosperi

**Affiliations:** ^1^Department of Epidemiology, University of Florida, Gainesville, FL, United States; ^2^Emerging Pathogens Institute, University of Florida, Gainesville, FL, United States; ^3^Department of Computer & Information Science & Engineering, University of Florida, Gainesville, FL, United States; ^4^Department of Veterinary Population Medicine, University of Minnesota, St. Paul, MN, United States

**Keywords:** relative solvent accessibility, antimicrobial resistance, scoring, AMR, secondary structure, protein variant, RSA

## Abstract

Antimicrobial resistance (AMR) is a significant and growing public health threat. Sequencing of bacterial isolates is becoming more common, and therefore automatic identification of resistant bacterial strains is of pivotal importance for efficient, wide-spread AMR detection. To support this approach, several AMR databases and gene identification algorithms have been recently developed. A key problem in AMR detection, however, is the need for computational approaches detecting potential novel AMR genes or variants, which are not included in the reference databases. Toward this direction, here we study the relation between AMR and relative solvent accessibility (RSA) of protein variants from an *in silico* perspective. We show how known AMR protein variants tend to correspond to exposed residues, while on the contrary their susceptible counterparts tend to be buried. Based on these findings, we develop RSA-AMR, a novel relative solvent accessibility-based AMR scoring system. This scoring system can be applied to any protein variant to estimate its propensity of altering the relative solvent accessibility, and potentially conferring (or hindering) AMR. We show how RSA-AMR score can be integrated with existing AMR detection algorithms to expand their range of applicability into detecting potential novel AMR variants, and provide a ten-fold increase in Specificity. The two main limitations of RSA-AMR score is that it is designed on single point changes, and a limited number of variants was available for model learning.

## Introduction

Antimicrobial resistance (AMR) is a significant and growing public health threat. Treating infections caused by resistant organisms is clinically challenging, and sometimes impossible; even when resistant infections can be treated with alternative antibiotics, these treatments are often costly both in terms of healthcare costs as well as increased morbidity in treated patients. For these same reasons, AMR is a significant challenge in veterinary and plant health, and the need to develop novel AMR treatments is deemed very urgent (Nelson et al., 2019). According to a 2019 report (Centers for Disease Control and Prevention (U.S.), 2019), the number of annual AMR infections in the US alone is greater than 2.8 million, with 35,000 AMR-associated deaths. The CDC identifies 21 resistant bacteria and fungi as threats to human and public health, with 5 of those listed as urgent threats and 11 as serious threats. Efficient identification of AMR is of pivotal importance in order to control the spread of AMR and contain its impact. To address this need, several AMR databases and identification algorithms have been recently developed, including MEGARes (Doster et al., 2020) and CARD (Alcock et al., 2020). Curated records in AMR databases typically list whole resistant gene accessions, i.e., genes resistant to specific molecules or AMR classes/mechanisms; or housekeeping genes associated with specific AMR-conferring amino acid variants. These AMR variants can originate from two main sources: (a) specific genes or mobile elements (e.g., plasmids) conferring AMR; or (b) specific protein variants that connote AMR in housekeeping regions. Hundreds of variants are known to confer resistance. The MEGARes database, for example, reports about 500 of these mutations, with the five more frequent mechanisms being Fluoroquinolones (110), Rifampin (46), Phenicol (40), Sulfonamides (39), and Aminocoumarins (37). There is a continuous, active search performed by the scientific community to discover genomic variants conferring or altering AMR in bacteria strains, and to assess their prevalence in isolates. For example, specific variants on ParC gene, such as S83I, D87G, and GyrA gene, such as C257T, D99N, G93C, and M95I, confer resistance to fluoroquinolone. Recent works on these variants include studies on *Mycoplasma genitalium* from urethral swabs or urine sediments (Hamasuna et al., 2018); *Neisseria gonorrhoeae* from urethral or cervical swabs (Kivata et al., 2019); *Klebsiella pneumonia* from blood, wound, and sputum (Zeng et al., 2020); and *Campylobacter jejuni* from human diarrheal cases (Elhadidy et al., 2020).

**FIGURE 1:**
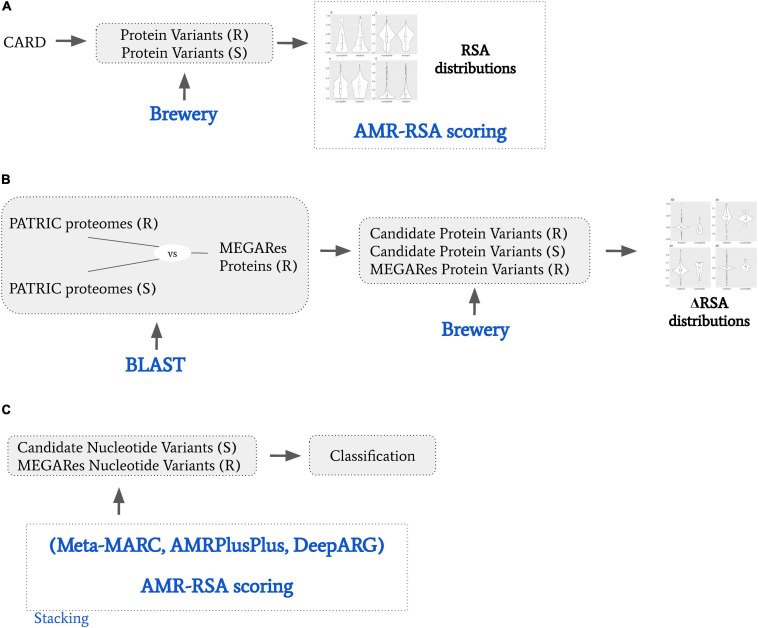
General schematic of the presented work. **(A)** We obtained from CARD resistant (R) and susceptible (S) protein variants. We used Brewery software to calculate relative solvent accessibility (RSA), and derived a RSA scoring model to dichotomize R and S variants based on their RSA scores. **(B)** We obtained from PATRIC proteomes that are resistant or susceptible to specific AMR molecular mechanisms. We BLAST searched them against MEGARes proteins resistant to the same AMR molecular mechanisms, and obtained candidate R and S protein variants. We then calculated the RSA variation (ΔRSA) by comparing candidate R and S protein variants against their MEGARES. **(C)** We considered S and R protein variant pairs by coupling the candidate susceptible protein variants with their MEGARes counterparts. We classified them by stacking Meta-MARC, AMRPlusPlus, and DeepARG to the RSA scoring model; and we designed a meta-feature ensemble approach.

Systems for automated AMR identification such as AMRPlusPlus (Doster et al., 2020), Meta-MARC (Lakin et al., 2019), or DeepARG (Arango-Argoty et al., 2018), are intrinsically based on their reference databases, which are highly incomplete. In other words, current identification methods are limited to only genes and pathogens in the reference databases. The automatic identification of novel AMR genes remains therefore an open problem. The wetlab methods to discover a new AMR gene are highly laboratory intensive, requiring specifically trained personnel to be performed. As resources are limited, an automated system discovering new potential AMR genes would be beneficial to isolate highly plausible, testable AMR candidates. With the present study we take an initial step in this direction by developing an AMR scoring system based on protein secondary structure variations, focusing on relative solvent accessibility (RSA). The relative solvent accessibility of a residue in a peptide is the fraction of the surface area that is accessible to a solvent, and reflects the level of exposure of the residue. An exposed residue is more likely to interact with the surrounding environment than a buried one. A variation in the solving accessibility in a key AMR residue would therefore likely affect the AMR machinery. Structure variation helps understanding the impact of mutations in drug resistance (Pandurangan et al., 2017). Exposed protein residues tend to contribute less to protein stability and evolve faster (Ramsey et al., 2011). A variation in the solving accessibility in a key AMR residue would therefore likely affect the AMR machinery. Examples of variation of secondary structure affecting AMR include alteration of beta-lactamase resistance (Liu et al., 2019); cephalosporins, meropenem, imipenem, ciprofloxacin, chloramphenicol, cefepime, cefotetan, cefotaxime, cefpirome, and ceftazidime (Doménech-Sánchez et al., 2003); multi-compound, RND efflux regulator resistance (Jahandideh, 2013); isonazid resistance (Purohit et al., 2011); and macrolide resistance implied by conserved protein structure (Stsiapanava and Selmer, 2019). In this work (a) we find how RSA consistently characterizes known CARD resistant and susceptible variants, with resistant variants more likely to be exposed, and susceptible variants more likely to be buried; (b) we show how the variants in AMR MEGARes proteins with the potential of hindering the resistance mechanism tend to be denoted by RSA variation; (c) we elaborate an AMR protein variant scoring system based on RSA (RSA-AMR score), and we show it can be used to expand the range of applicability of an existing AMR detection algorithm. A general schematic describing our procedure is represented in Figure 1.

## Materials and Methods

### Relative Solvent Accessibility Calculation

Briefly, Brewery (Torrisi and Pollastri, 2020) is a state-of-the-art deep learning method implementing stacked bidirectional recurrent neural networks and convolutional neural networks to predict protein secondary structure, considering evolutionary information from PSI-BLAST (Altschul et al., 1997) and HHblits (Remmert et al., 2012). We considered the prediction of relative solvent accessibility (RSA), i.e., the exposed fraction of the maximum possible solvent accessible surface area for a residue. The input for Brewery is the protein sequence, while the output is the probability distribution of each residue of belonging to four exposure classes, namely *B*, *b*, *e*, and *E*. The classes are described as follows: *B*, very buried (less than 4% exposed); class *b*, somewhat buried (between 4 and 25% exposed); class *e*, somewhat exposed (between 25 and 50% exposed); and class *E*, very exposed (more than 50% exposed). For each residue, the scores predicted for all the classes represent a probability distribution, therefore their probability sum is bound to be 1.

### CARD AMR Protein Variants

We extracted the protein variants from the CARD protein variant model, considering single resistance variants only. To describe the variant, CARD reports position, susceptible (wild type) amino acid, and resistant one. For example, a record of a resistance variant for *Bacillus subtilis pgsA* with mutation conferring resistance to daptomycin is annotated as A64V, meaning that a Valine in position 64 confers the described resistance. In addition to the variant annotation, CARD reports the susceptible protein sequence as well. For our analysis, if the susceptible residue in the annotated position differed from the one reported in the sequence, we considered both as susceptible. This procedure led to the collection of 1,234 resistant protein variants out of 152 susceptible proteins.

These data are used firstly to measure the difference in the RSA classes between resistant and susceptible variants, and then to elaborate the RSA-AMR score.

### Candidate AMR Protein Variants From MEGARes and PATRIC

CARD AMR protein variants typically belong to housekeeping genes. To extend our study to a more general case, we extracted candidate AMR and susceptible protein variants by aligning AMR MEGARes proteins with both AMR and susceptible proteomes downloaded from PATRIC (Davis et al., 2020). We selected MEGARes genes annotated to be resistant to a specific AMR molecule or mechanism. We then selected matching PATRIC proteomes that are annotated as either resistant or susceptible to the same AMR molecule or mechanism. After translating the MEGARes genes to proteins, we aligned the MEGARes proteins against PATRIC proteomes with BLAST, retaining extensive and precise alignments (see below). We selected the resulting BLAST alignment mismatches as candidate new variants. Variants found in the alignment of MEGARes (resistant) with PATRIC susceptible proteins are candidates to be responsible for the susceptibility. That is, if a MEGARes protein is *resistant* to a specific AMR molecule or mechanism, and it is well and extensively aligned against a PATRIC protein from a proteome which is *susceptible* to the same AMR molecule or mechanism, then the alignment mismatches can be read as candidate variants involved in the resistance loss. Conversely, candidate variants (i.e., alignment mismatches) found in proteins from PATRIC resistant proteomes are more likely to not affect the resistance, as both the MEGARes and PATRIC proteins are annotated with the same AMR resistance.

### MEGARes and PATRIC Data Extraction and Alignment

MEGARes V2.00 genomic sequences were obtained and AMR variant-dependant accessionsed were filtered out (7375 sequences retained). We translated MEGARes sequences into peptides with ORFfinder (Rombel et al., 2002), considering any sense codon (other parameters set to default). PATRIC proteomes were filtered according to specific annotations, as follows. Genomes metadata were downloaded from the PATRIC website (415089 annotations). We removed all annotations that were not explicitly listed as “Resistant,” “Susceptible,” “Intermediate,” “Non-susceptible,” “S,” or “RS,” and did not have an explicit testing standard recorded (135198 retained annotations). As PATRIC provides proteomes, we did not translate PATRIC genomes, but used the provided proteomes.

We then searched for specific terms to extract drug-specific resistant MEGARes sequences, and drug-specific resistant and susceptible PATRIC proteomes, namely tetracyclines, ciprofloxacin, ampicillin, and amikacin. Our rationale for choosing the PATRIC labels used in this work was based on three criteria, namely (a) abundance of the labels in both databases; (b) one-to-one non-ambiguous mapping between MEGARes hierarchical nomenclature and PATRIC labels; and (c) labels that confer resistance to antimicrobials listed as critically important for human medicine by World Health Organization [WHO] (2019). Class A beta-lactamases have 1580 entries in MEGARes, and ampicillin is the most frequent beta-lactamase label in both susceptible (3994) and resistant (4424) filtered PATRIC records. Aminoglycosides have 773 entries in MEGARes, and amikacin is among the most frequent aminoglycoside labels in both susceptible (3531) and resistant (821) filtered PATRIC records. Fluoroquinolones have 296 entries in MEGARes, and ciprofloxacin is the most frequent fluoroquinolone label in both susceptible (3995) and resistant (4221) filtered PATRIC records. Tetracyclines have 296 entries in MEGARes, and tetracyclines are the most frequent AMR class label in both susceptible (3354) and resistant (4089) filtered PATRIC records. Tetracyclines are the most abundant in PATRIC, and directly correspond to an existing MEGARes term. In its 2018 list of critically important antimicrobials, WHO classifies tetracyclines as important, and fluoroquinolones, beta-lactamases, and aminoglycosides as critical. Considering each specific term independently, we filtered out PATRIC proteomes that were annotated as both susceptible or resistant to that term. For tetracyclines, we retained PATRIC genomes if annotated with the terms “tetracycline,” or “tetracyclin,” and MEGARes genes if annotated as tetracyclines. For ciprofloxacin, we retained PATRIC proteomes if annotated with the term “ciprofloxacin,” and MEGARes genes if annotated as fluoroquinolones. For ampicillin, we retained PATRIC proteomes annotated with the term “ampicillin,” and MEGARes genes if annotated as Class A betalactamases. For Amikacin, we retained PATRIC proteomes annotated with the term “amikacin,” and MEGARes sequences if annotated with as aminoglycosides. Once obtained the PATRIC proteomes, we generated two BLAST databases (DBs) for each molecule, one with resistant proteomes, one with susceptible ones. We then BLAST searched the selected MEGARes peptides against their paired DBs, obtaining two sets of alignments, one against resistant proteomes, one against susceptible ones. From these two DBs we extracted, respectively, candidate resistant and candidate susceptible protein variants as the alignment mismatches. We considered as candidate variants the single mismatches in BLAST ungapped alignments with an identity of 99% or more, extending for 80% or more of the MEGARes query peptide. For tetracyclines we retrieved 346 resistant and 24 susceptible variants from 83 MEGARes proteins. For ciprofloxacin, we retrieved 76 resistant and 72 susceptible variants from 135 MEGARes proteins. For ampicillin, we retrieved 1833 resistant and 189 susceptible variants from 889 MEGARes proteins For amikacin, we retrieved 150 resistant and 156 susceptible variants from 294 MEGARes proteins.

### RSA Class *P*-Values

For CARD protein variant RSA probability distributions, *P*-values comparing the score distributions have been calculated as pairwise (resistant versus susceptible) Wilcoxon signed-rank tests. *P*-values for probability distributions from candidate protein variants derived from MEGARes and PATRIC are calculated as Wilcoxon rank sum tests.

### Gene Data Set to Test the Combination of RSA-AMR Score With Existing AMR Algorithms

To show how the RSA-AMR score can ameliorate the performance of existing AMR detection algorithms, we used it in combination with Meta-MARC, DeepARG, and AMRPlusPlus. We obtained a set of resistant-susceptible gene pairs as follows. We considered all the candidate susceptible protein variants derived by aligning MEGARes versus PATRIC, as in Sections 2.3 and 2.4. For each susceptible protein variant, we calculated all the possible corresponding nucleotide codons, and obtained a candidate susceptible gene for each codon. We coupled candidate susceptible protein variants with their reference resistant gene on MEGARes obtaining a balanced data set of resistant and candidate susceptible instances. Regarding classification for Meta-MARC, we considered the lowest E-score for each sequence; for DeepARG, we considered the DeepARG AMR probability output for each sequence; for AMRPlusPlus we considered the resistome, i.e., the sum of the AMRPlusPlus BWA alignements to the reference database.

## Results

### *In silico* RSA Is Increased in CARD AMR Protein Variants

**FIGURE 2:**
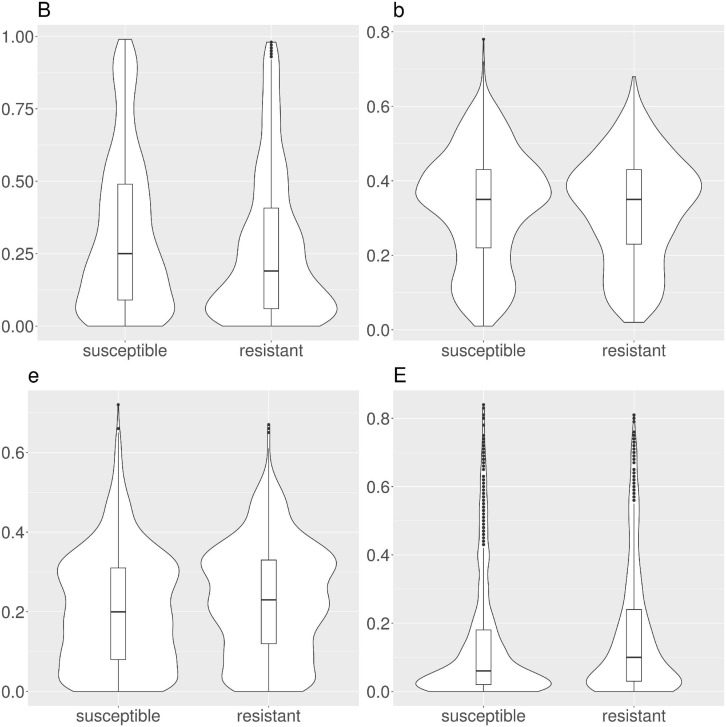
Violin and box plots for the solvent accessibility scores in CARD protein variants. Resistant variants are significantly less buried (class *B*), and more exposed (classes *e* and *E*).

We calculated the solvent accessibility for each protein, susceptible and resistant, from 1,234 resistant protein variants obtained from the CARD (Table 1 and Figure 2). The Brewery algorithm (Torrisi and Pollastri, 2020) outputs the probability of each residue to belong to four exposure classes, namely *B* (very buried), *b* (somewhat buried), *e* (somewhat exposed), and *E* (very exposed). We found resistant variants show a higher probability than susceptible ones in classes *e* (medians 0.23 versus 0.2), and *E* (medians 0.24 versus 0.06). Conversely, susceptible protein variants show a higher probability in class *B* when compared to resistant ones (medians 0.25 and 0.19, respectively). We did not find a variation in the medians for class *b* (both 0.35).

**TABLE 1:** Interquartile range and *p*-values comparing the scores of susceptible and resistant protein variants for each of the four RSA classes.

	**Susceptible**			**Resistant**			
	**1st Q**	**Median**	**3nd Q**	**1st Q**	**Median**	**3rd Q**	**Pval**
*B*	0.09	0.25	0.49	0.06	0.19	0.4	2.2e-16
*b*	0.22	0.35	0.33	0.23	0.35	0.43	0.092
*e*	0.08	0.2	0.31	0.12	0.23	0.33	2.2e-16
*E*	0.02	0.06	0.18	0.03	0.1	0.24	2.2e-16

We investigated the solvent accessibility difference between susceptible and resistant variants across specific AMR mechanisms (Supplementary Figures 1–6). We retained the six CARD model types with at least 30 annotated susceptible/resistance pairs, namely one *Clostridioides difficile* resistance to fluoroquinolone, and six *Mycobacterium tuberculosis* resistances (ethambutol, streptomycin, isoniazid, pyrazinamide, and rifampicin). We found the RSA behavior to be consistent also if we split the variants by mechanism. Class *B* is invariantly scoring higher for susceptible protein variants, while classes *e* and *E* for resistant ones. Differently from the general case, class *b* is scoring higher for susceptible protein variants in four out of six molecular mechanisms.

### AMR RSA Score

**FIGURE 3:**
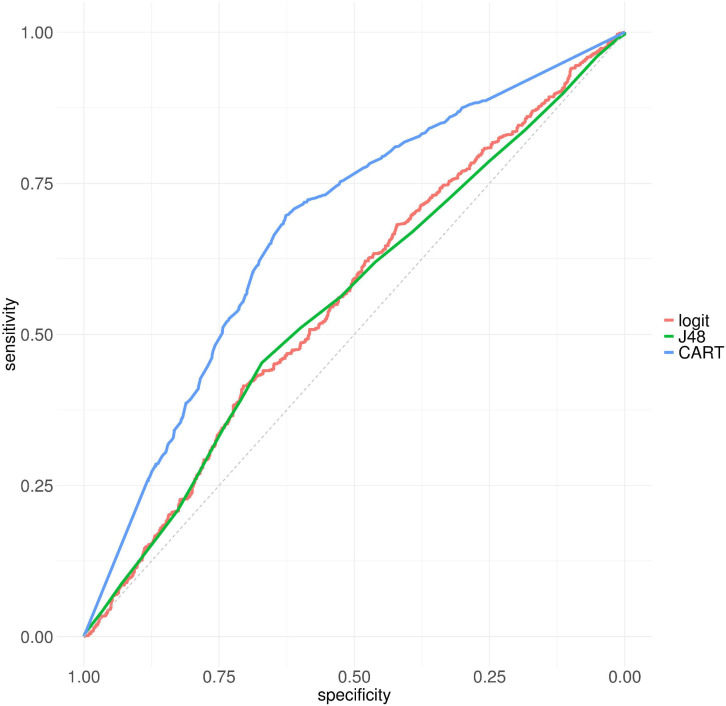
ROC curves for logistic regression, J48, and CART on CARD protein variant data set, 10 fold cross-validation.

By considering resistant and susceptible CARD AMR variants as classes, we developed a model based on logistic regression, with the four RSA values as features. We used the probability of this model as a RSA-based AMR protein variant score (RSA-AMR score). We measured the area under the ROC curve (AUC) for a 10-fold cross validation for logistic regression (AUC 0.55), J48 decision tree (AUC 0.55), and CART (AUC 0.67) decision tree, as depicted in Figure 3. CART provided the best AUC but at the cost of an increased complexity (number of leaves 247, tree size 493) compared to J48 (number of leaves 11, tree size 21).

### *In silico* Candidate AMR Protein Variants From MEGARes and PATRI*C*

We extracted candidate AMR and susceptible protein variants by aligning AMR MEGARes proteins with both AMR and susceptible PATRIC proteomes.

When examining CARD variants, we use two sets of RSA probability distributions, i.e., the ones for the susceptible (wild type) variants, and the ones for the resistant variants. For candidate variants extracted from the MEGARes versus PATRIC alignment, on the other hand, we have to consider three sets of RSA probability distributions. Specifically, we calculate the RSA for the resistant (original) residues from MEGARes; the RSA for the candidate resistant variants; and the RSA for the candidate susceptibility variants. We therefore do not compare RSA probability distributions but *variations* of the RSA probability distributions (Δs), as the difference between the resistant (MEGARes) solvent accessibility distribution; and the candidate resistant or susceptible variant (PATRIC) solvent accessibility distribution. Δ*B*, Δ*b*, Δ*e*, and Δ*E* are calculated as MEGARes residue score (resistant) – PATRIC score. Therefore when we consider genes from susceptible PATRIC genomes, we expect a change in the score; whereas when we consider genes from resistant PATRIC genomes, we expect a negligible score change, ideally Δ*B*, Δ*b*, Δ*e*, and Δ*E* = 0.

**FIGURE 4:**
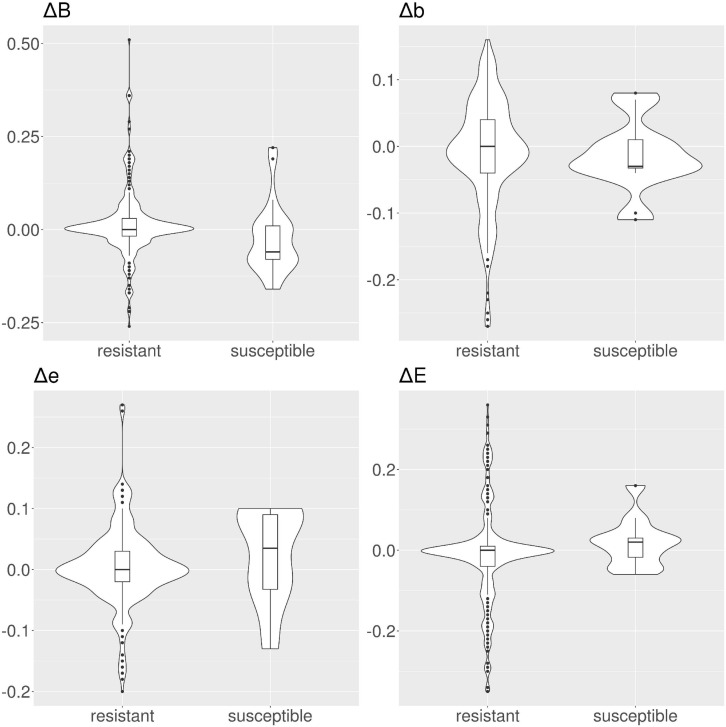
Violin and box plots for the solvent accessibility scores for tetracyclines. For resistant protein variants, Δs for all classes have a median of 0, showing no variation in solvent accessibility. On the contrary, susceptible protein variants show median Δ≠0.

**FIGURE 5:**
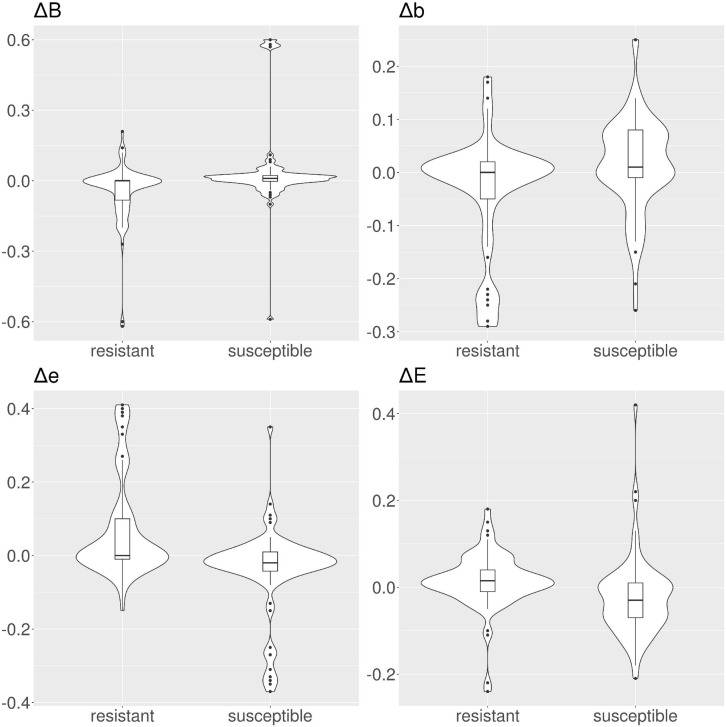
Violin and box plots for the solvent accessibility scores for ciprofloxacin. For resistant protein variants, with the exception of class *E*, Δs have a median of 0, showing no variation in solvent accessibility. On the contrary, susceptible protein variants Δ with median ≠ 0.

**FIGURE 6:**
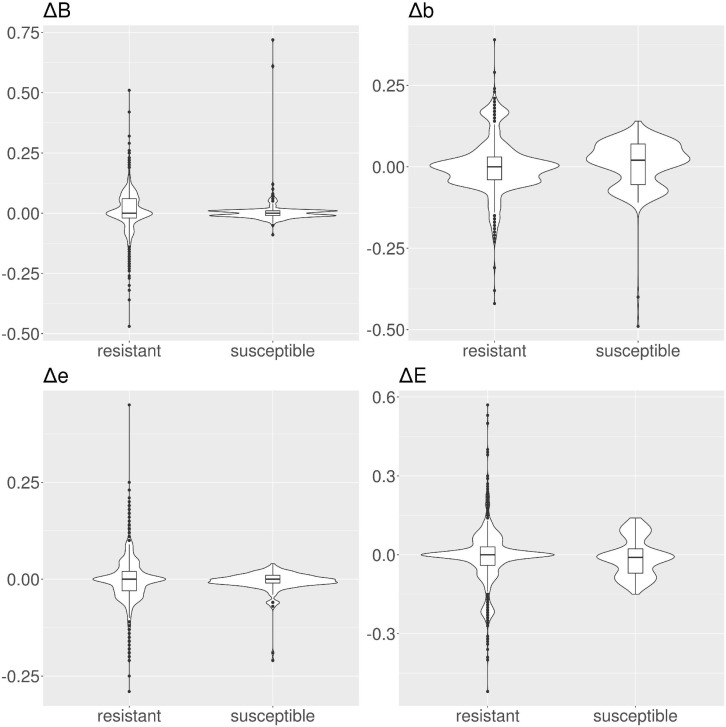
Violin and box plots for the solvent accessibility scores for ampicillin. For resistant protein variants, all Δs have a median of 0, showing no variation in solvent accessibility. Susceptible protein variants for Δ*b* and Δ*e* show median ≠ 0.

**FIGURE 7:**
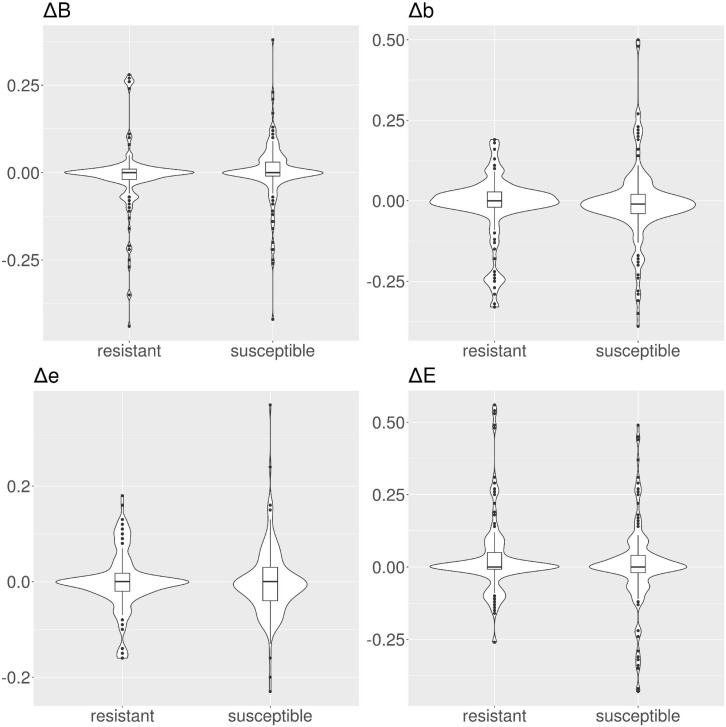
Violin and box plots for the solvent accessibility scores for amikacin. The median s for all classes are 0 for resistant and susceptible protein variants is 0, only for class Δ*b* in the susceptible protein variants the median is ≠ 0.

In tetracyclines (Table 2 and Figure 4), resistant protein variants Δs for all classes have median = 0, showing no variation in solvent accessibility. This indicates that PATRIC candidate resistant variants do not influence the solvent accessibility of the MEGARes resistant genes. On the contrary, PATRIC susceptible protein variants show median Δ≠0, suggesting a structural variation. In ciprofloxacin (Table 2 and Figure 5), resistant protein variants showing median Δ = 0, with the exception of class *E*, where the median Δ is negative. For susceptible protein variants, three out of four classes (*B*, *e*, and *E*) show Δ≠ 0, suggesting a structural variation. In ampicillin (Table 2 and Figure 6), the median Δs for all classes are 0 for resistant proteins; for the susceptible one, Δb and ΔE distributions are centered in 0, while ΔB and Δe are not. In amikacin (Table 2 and Figure 7), while the median Δs for all classes are 0 for resistant protein, only for class Δ*b* the median is ≠ 0 in the susceptible protein variants. Overall, these results suggest that even for genes without known resistance-conferring variants, conservation of the structural variations (or its absence) can be implicitly related to their ability to retain (or lose) AMR against specific substances.

**TABLE 2:** Interquartile ranges and *p*-values comparing the scores of candidate susceptible and resistant protein variants obtained by alignment mismatches of MEGARes proteins against PATRIC proteomes, against their resistant (original) MEGARes counterparts.

	**Susceptible**			**Resistant**			
	**1st Q**	**Median**	**3rd Q**	**1st Q**	**Median**	**3rd Q**	**Pval**
**Tetracyclines**							
Δ*B*	−0.08	−0.06	0.01	−0.017	0	0.03	0.0064
Δ*b*	−0.032	−0.03	0.01	−0.04	0	0.04	0.2796
Δ*e*	−0.03	0.035	0.09	−0.02	0	0.03	0.1328
Δ*E*	−0.017	0.02	0.03	−0.04	0	0.01	0.0707
**Ciprofloxacin**							
Δ*B*	−0.002	0	0.022	−0.082	0	0	< 0.0001
Δ*b*	−0.01	0.01	0.08	−0.05	0	0.02	0.0075
Δ*e*	−0.042	−0.02	0.01	−0.01	0	0.069	< 0.0001
Δ*E*	−0.07	−0.03	0.01	−0.01	0.015	0.04	< 0.0001
**Ampicillin**							
Δ*B*	−0.01	0	0.01	−0.02	0	0.06	0.0094
Δ*b*	−0.055	0.02	0.07	−0.04	0	0.03	0.0004
Δ*e*	−0.01	0	0.01	−0.03	0	0.02	0.5435
Δ*E*	−0.07	−0.1	0.02	−0.04	0	0.03	0.0157
**Amikacin**							
Δ*B*	−0.01	0	0.03	−0.02	0	0.01	0.02
Δ*b*	−0.04	−0.01	0.02	−0.02	0	0.027	0.1791
Δ*e*	−0.04	0	0.03	−0.02	0	0.017	0.2588
Δ*E*	−0.02	0	0.04	−0.007	0	0.05	0.5523

### Combining the SRA-AMR Score With AMR Detection Algorithms

To show how it is possible to utilize the RSA-AMR score to expand the range of applicability of existing AMR detection algorithms, we show three case studies with state-of-the-art algorithms, namely Meta-MARC, DeepARG, and AMRPlusPlus. We derived a set of resistant and susceptible genes by pairing candidate susceptible variants described in Section 2.6. We chose this set as we can not use CARD since the RSA-AMR score is based on those very same variants. Resistant genes from Megares are paired with candidate susceptible ones, i.e., carrying a variant obtained from PATRIC alignments. Current algorithms are not designed to answer the specific task represented by our data set, which would be to detect susceptible sequences from their resistant counterparts based on a single protein variant per sequence, as their AMR labeling is based on sequence similarity (for example percent of identity with sequences in the reference database). We observed that these algorithms classify the great majority of the candidate susceptible variants as resistant (false positives). We show how using the SRA-AMR score can greatly ameliorate the results and greatly curb the number of false positives, increasing specificity.

**FIGURE 8:**
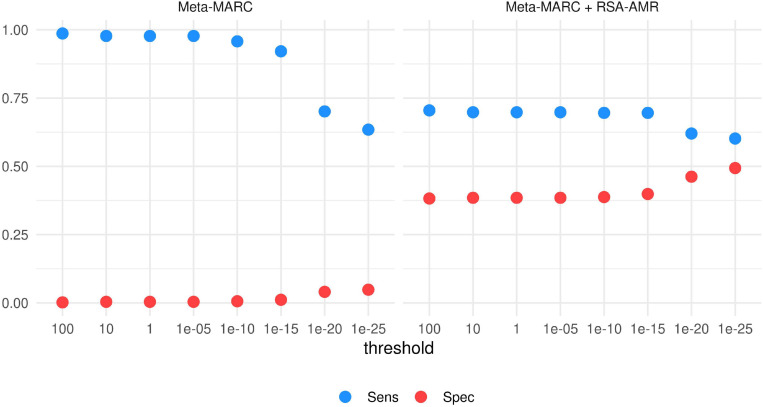
Sensitivity and Specificity of Meta-MARC and Meta-MARC combined with the RSA-AMR score. The best combination of Sensitivity and Specificity is achieved by combining Meta-MARC combined with the RSA-AMR score, and a stringent threshold of 10^– 25^ for the Meta-MARC E-value.

**Integration with Meta-MARC.** The Meta-MARC predicted resistome depends on a user-defined E-value threshold. To explore the integration of the RSA-AMR score with Meta-MARC, we tested how Sensitivity and Specificity vary according to the E-value threshold, and how the RSA-AMR integration can ameliorate the overall performance (Figure 8). We filtered the paired variants to retain unique variants only (437 resistant and 1519 susceptible variants). We tested E-value thresholds of 100, 10, 1, 10^–5^, 10^–10^, 10^–15^, 10^–20^, and 10^–25^. Meta-Marc tends to identify the great majority of the variants as resistant for threshold 10^–15^ and above, with Sensitivity ≥0.92 and Specificity ≤0.01. For thresholds below 10^–15^, Specificity shows a slight increase (up to 0.05 for E-value 10^–25^), but at the cost of a sharp decline in Sensitivity (down to 0.63 for E-value 10^–25^). Thus, Meta-MARC cannot accurately distinguish genes carrying the candidate susceptible variant from the resistant ones, as it tends to classify both classes as resistant. We used the RSA-AMR score to balance Sensitivity and Specificity in an ensemble (stacking) fashion. We considered the Meta-MARC predictions for the resistant class as true; however, for the susceptible class, we considered the Meta-MARC predictions true only if the associated RSA-AMR score is positive, i.e., predicted positive by the RSA-AMR logistic regression. When compared to the Meta-MARC prediction for the most stringent threshold of 10^–25^, Specificity is greatly increased (0.05–0.49), with a minor loss of Sensitivity (0.63–0.6).

**FIGURE 9:**
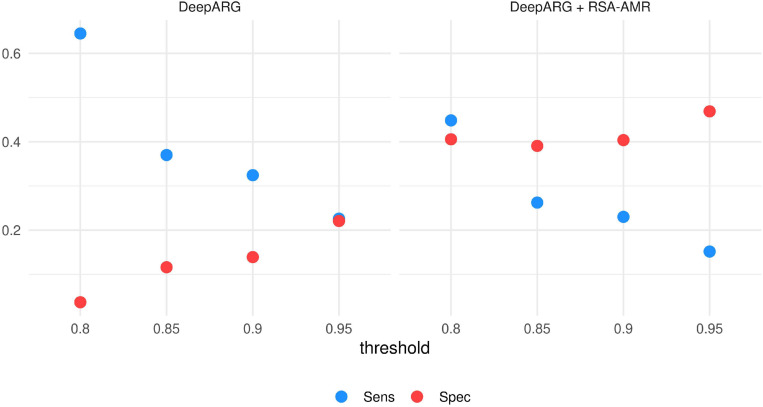
Sensitivity and Specificity of DeepARG and DeepARG combined with RSA-AMR. The best combination of Sensitivity and Specificity is achieved by combining DeepARG with the RSA-AMR score, and the default 0.8 threshold for DeepARG.

**Integration with DeepARG.** The same data set utilized for the Meta-MARC integration was also tested with DeepARG. We varied the DeepARG classification threshold from 0.8 (default) to 0.95 (Figure 9). At default threshold value, DeepARG tends to classify the majority of the variants as resistant, with Sensitivity 0.65 and Specificity 0.04. By raising the threshold, Specificity increases up to 0.23, but Sensitivity sharply drops to 0.22. By adding the RSA-AMR score to the DeepARG classification, we observe a similar behavior, with Specificity increasing and a Sensitivity decreasing as the threshold is raised; however, the default DeepARG threshold combined with the RSA-AMR score provides the best combination of Sensitivity (0.45) and Specificity (0.4).

**Integration with AMRPlusPlus**. To make possible an integration with AMRPlusPlus, we proceeded by providing AMRPlusPlus the unfiltered paired resistant/candidate susceptible sequences and their reverse complement, and setting the highest quality for all the corresponding simulated FASTQ files to ensure that AMRPlusPlus would not filter out any sequence. Not surprisingly, the total number of BWA alignments is basically the same for both classes, with 3587 and 3585 BWA alignments for resistant and candidate susceptible classes respectively. We can apply the RSA-AMR score to curb the tendency of AMRPlusPlus to find the same resistome for both classes; specifically, we can pre-filter the sequences to be used as AMRPlusPlus by removing the ones with a negative RSA-AMR score, therefore less likely to carry resistance. By applying AMRPlusPlus to the filtered set, the number of alignments of the susceptible class (false negatives) decreases by 65% (1266 alignements), while the alignements on the resistant class (true positives) decreases only by 38% (2240 alignements).

**FIGURE 10:**
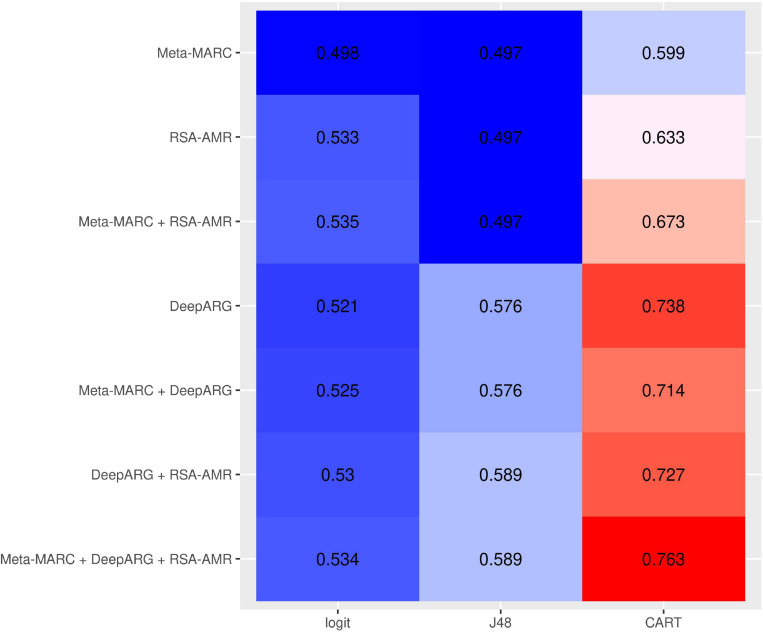
10-fold CV AUC for meta-classifiers based on different combinations of input meta-features and learning approaches. The best approach consists of a CART tree learning from all the three meta-features.

**RSA-AMR as a meta-classfier**. We assessed the potential of integrating Meta-MARC, DeepARG, and the RSA-AMR score in a meta-classifier. The meta-classifier is an AMR variant resistance classifier that accepts the outputs of single classifiers as input. We considered the filtered resistant/candidate susceptible data set to train meta-classifiers based on logistic regression, J48, and CART. As features for the meta-classifiers we considered the RSA-AMR score, the Meta-MARC E-value, and the DeepARG classification probability, or a combination of them. Figure 10 shows a heatmap of the 10-fold cross validation AUC for the different combinations of features and methods. Combining the meta-features from all the three approaches leads to the best results for all the learning methods.

## Discussion and Conclusion

The main state-of-the-art algorithms used to predict AMR in unknown sequences are based on alignment with known AMR genes (Arango-Argoty et al., 2018; Lakin et al., 2019; Alcock et al., 2020; Doster et al., 2020), and are therefore limited to search within their reference databases. While extremely functional, these algorithms can not be utilized to investigate novel AMR genes or variants. The need to fill this research gap motivated our work. Our analysis showed a strong RSA distribution shift related to the AMR-conferring protein variants (Figure 2 and Table 1), present in 3 out of 4 RSA classes. The direction of this shift is coherent with the fact that AMR is determined by structural physico-chemical properties, and therefore the AMR-related residues tend to be more exposed than their susceptible counterparts.

Consistently with these findings, we did not find a strong shift when measuring the ΔRSA in candidate resistant protein variants obtained by comparing resistant PATRIC proteomes and MEGARes resistant genes (median Δ = 0 for all the considered AMR mechanisms). On the other hand, the shift was present in the candidate susceptible protein variants, indicating a structural change. Intuitively, this change can be interpreted with the fact that a structural change is altering the AMR properties, specifically diminishing or removing AMR. However, the shift greatly varies according to the specific AMR mechanism (Figures 4–7 and Table 2) in terms of both classes (present in one class in amikacin; in all four classes in tetracyclines) and directions. In tetracyclines, susceptible variants tend to be more buried, showing negative Δ for classes *b* and *B*, and conversely positive Δ for classes *e* and *E*. However, this does not hold for other AMR mechanisms, where we notice a predicted structural variation in the candidate susceptible variants in both directions. There are several factors that, alone or combined, can explain why the shifts are in both directions. One possible explanation is that we use single-point mutations, whilst more extensive combinations of mutations could be needed for significant structural changes. However, when analyzing the so-called housekeeping genes that are known to confer AMR with exactly one mutation, we clearly observe a definite, strong shift in Δs between the E and B categories. Another possibility is that different antibiotics have different binding affinity and mechanisms of action, such that some might be less affected by structural changes, particularly if their mechanism of action doesn’t involve binding in that portion of the protein. Furthermore, the resistance mechanism for some proteins might in fact be due to burying of the binding site itself, i.e., by exposing a specific residue the resistance is hindered because the target antibiotic can bind. In the case of active translation, the protein blocks the binding site without preference for the direction of the change, depending on drug and mechanism. Finally, we are conducting *in silico* experiments based on predicted (not observed) data, i.e., predicted secondary structure and candidate protein variants. Therefore our data are strong enough to contain the biological signal connecting changes in solvent accessibility to changes in AMR, but are not sufficient to define its direction.

A challenge in our experiment design was to match CARD, MEGARes, and PATRIC annotations, which required *ad hoc* manual curation. While MEGARes provides a lookup table matching its sequences with the CARD ones, the AMR mechanism description is fundamentally different in the two databases. MEGARes structures each sequence into type, class, mechanism, and group; CARD provides an ontology-oriented labeling. PATRIC is instead annotated with AMR molecule-specific names. PATRIC annotations are not always consistent, with the same AMR molecule labeled with multiple names, or genomes showing records as both resistant and susceptible to the same AMR molecule (see Methods). Although designed for single variants, the RSA-AMR score can be applied in principle to proteins showing multiple variants, e.g., in an additive fashion. However, multiple non-synonymous changes can significantly impact the protein structure. In turn, structures computed by prediction tools might not be reliable when calculated upon multiple changes, possibly introducing larger errors. We plan to address a multiple-variant model in our future works.

In conclusion, in this study we analyzed AMR protein variants from the RSA perspective. We analyzed the RSA of AMR protein variants with Brewery, a state-of-the-art method based on deep learning. We found a strong distribution shift in resistant residues, with respect to RSA, when compared to the susceptible ones. Our approach unveiled RSA differences also in putative resistant and susceptible variants, obtained by aligning MEGARes and PATRIC sequences. These differences are measured as the variation in the RSA probability distribution. We found these differences varying greatly depending on the considered AMR machinery. Based on these findings, we developed a novel scoring system, RSA-AMR. RSA-AMR is not meant to be used as a standalone AMR prediction tool, but rather as a variant screening. When combined with state-of-the-art classifiers, it provides a ten-fold increase in predictive Specificity (combined with DeepARG, from 0.04 to 0.4; combined with Meta-MARC from 0.05 to 0.49). Limitations of RSA-AMR include the fact that it works on single point changes, and a limited number of identified protein variants was available for model learning.

## Data Availability Statement

The datasets generated and analyzed for this study can be found in the RSA-AMR github page. https://github.com/smarini/RSA-AMR.

## Author Contributions

MP, CB, SM, IBS, and NRN conceived the project. SM and MP designed the experiments. SM and MO conducted the experiments. SM, MP, and CB wrote the manuscript. All authors revised the manuscript.

## Conflict of Interest

The authors declare that the research was conducted in the absence of any commercial or financial relationships that could be construed as a potential conflict of interest.
