# Supplementary material for: Exploring Prediction of Antimicrobial Resistance Based on Protein Solvent Accessibility Variation
Source: Front Genet. 2021 Jan 22;12:564186. doi: 10.3389/fgene.2021.564186 (PMC7862766; doi:10.3389/fgene.2021.564186)
Supplement: Supplementary file 1 [file Data_Sheet_1.PDF]

Supplementary Material

1 Supplementary Figures

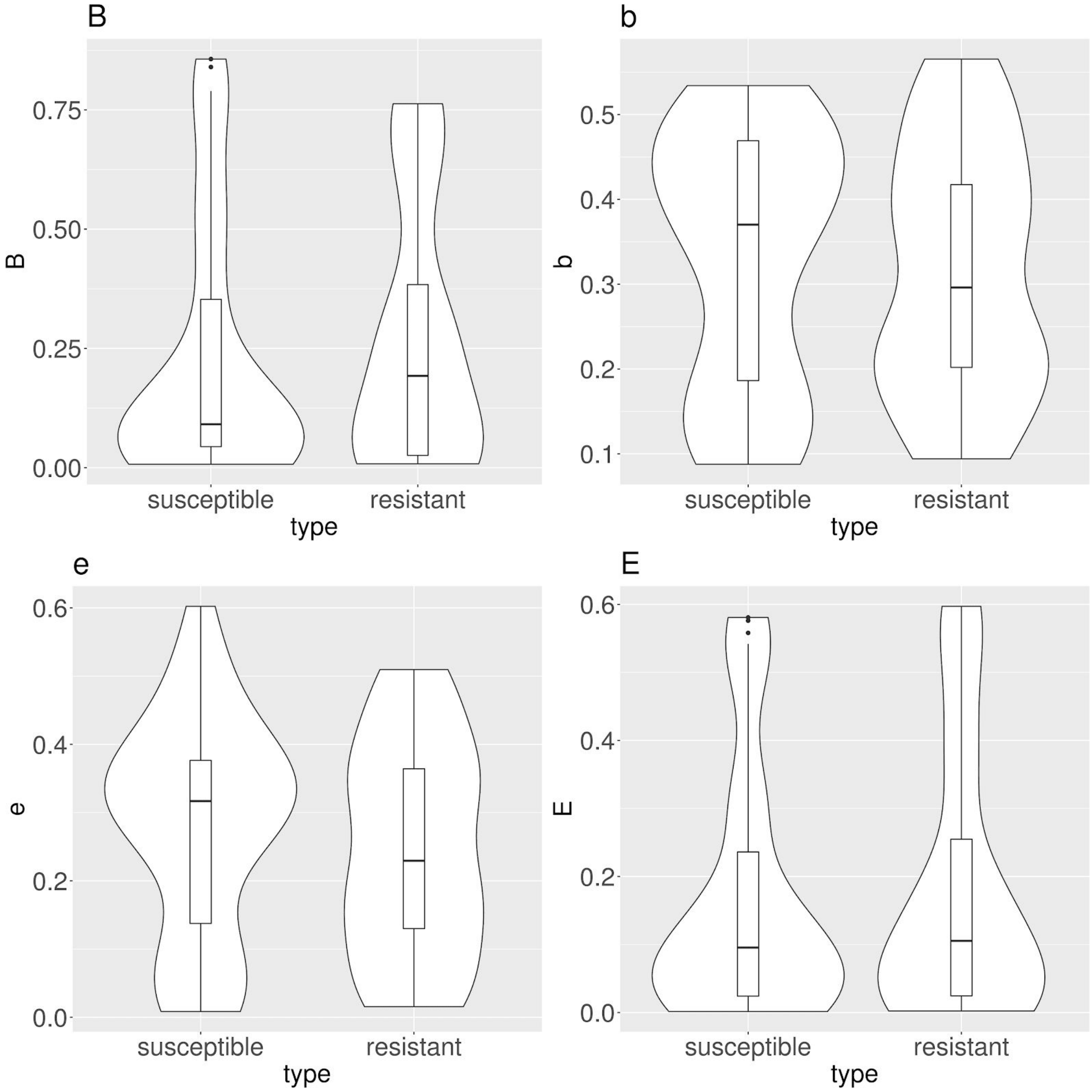

Supplementary Figure S1. Violin and box plots of the solvent accessibility scores for *Clostridioides difficile gyrB* conferring resistance to fluoroquinolone.

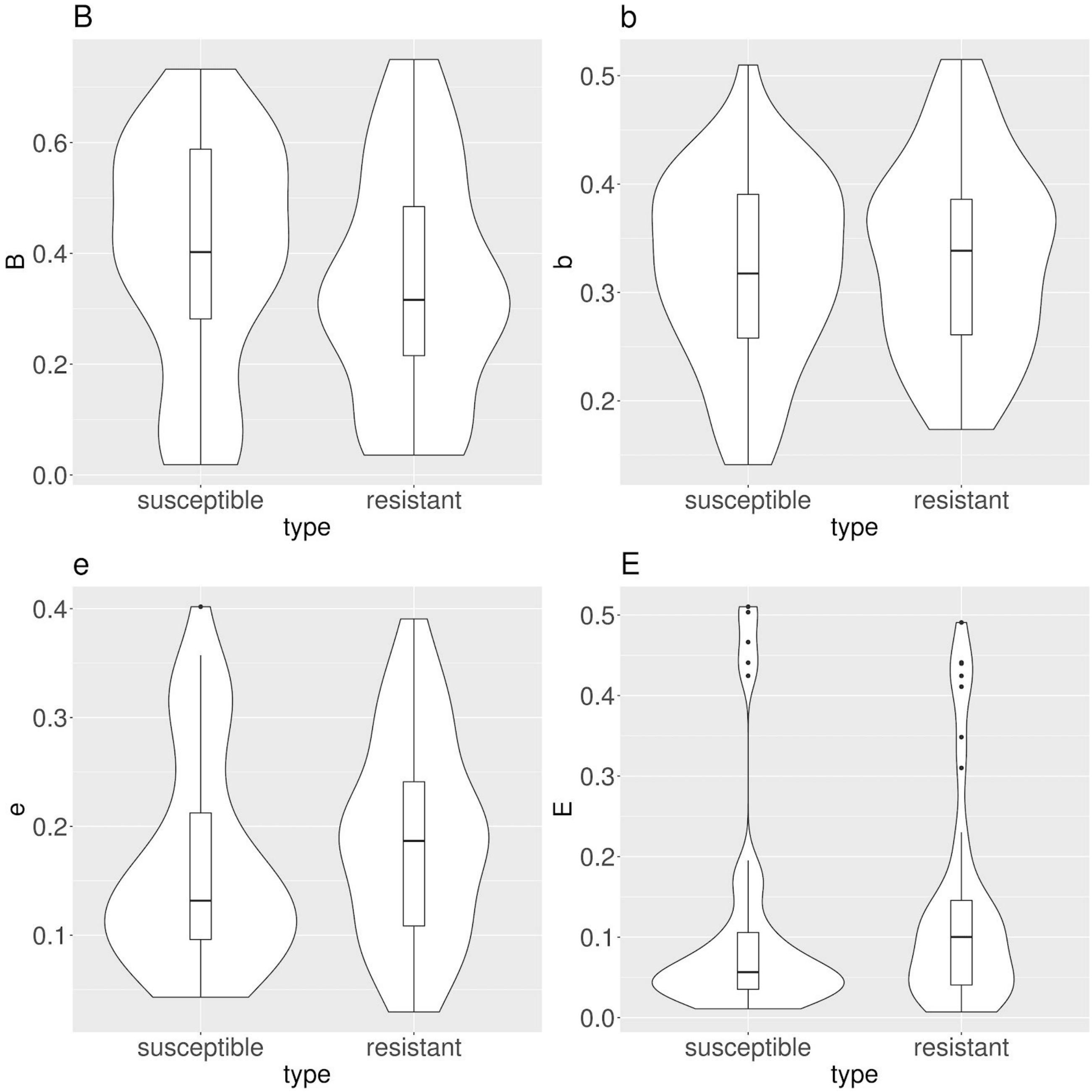

Supplementary Figure S2. Violin and box plots of the solvent accessibility scores for *Mycobacterium tuberculosis embB* with mutation conferring resistance to ethambutol.

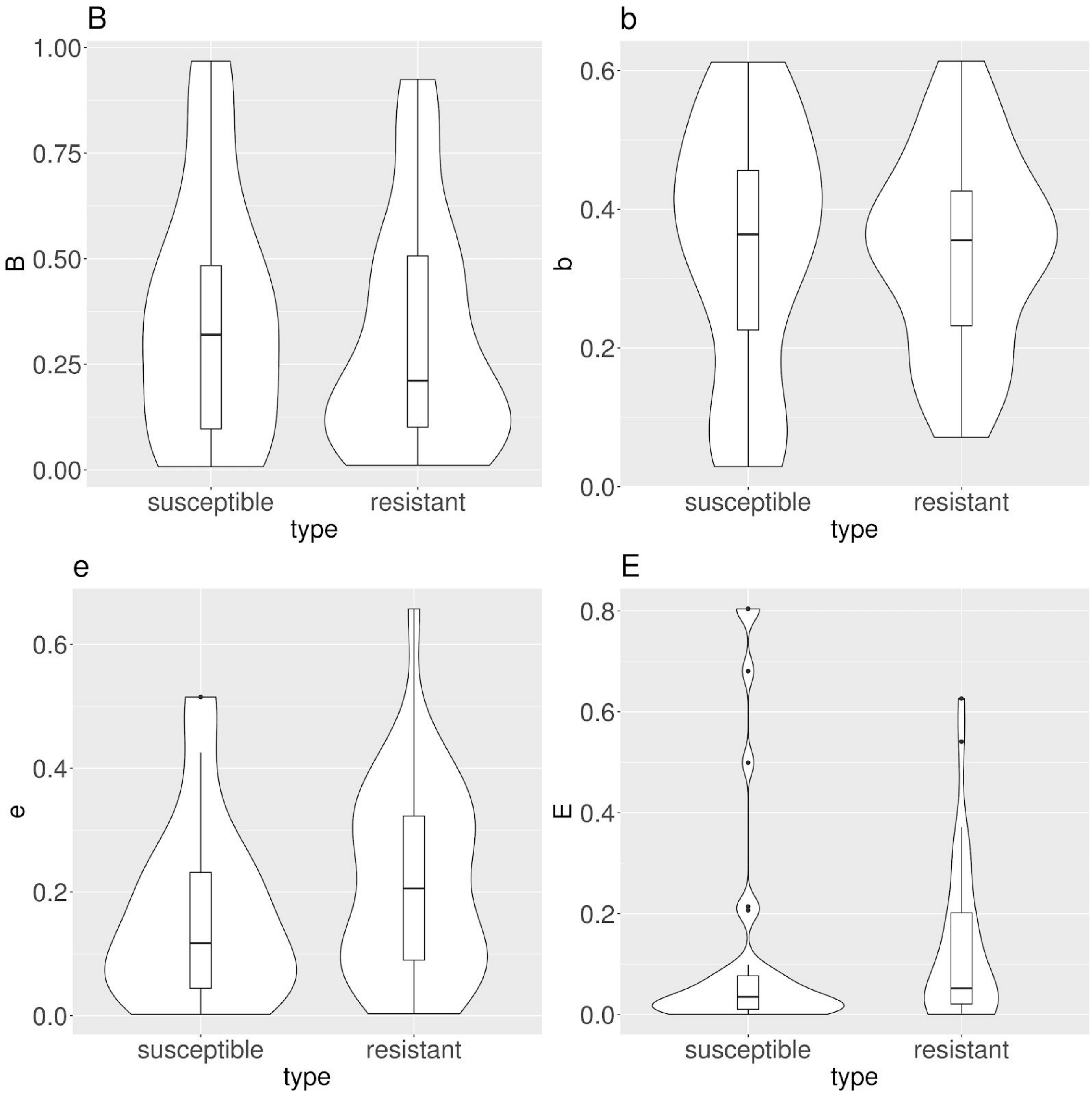

Supplementary Figure S3. Violin and box plots of the solvent accessibility scores for *Mycobacterium tuberculosis gidB* mutation conferring resistance to streptomycin.

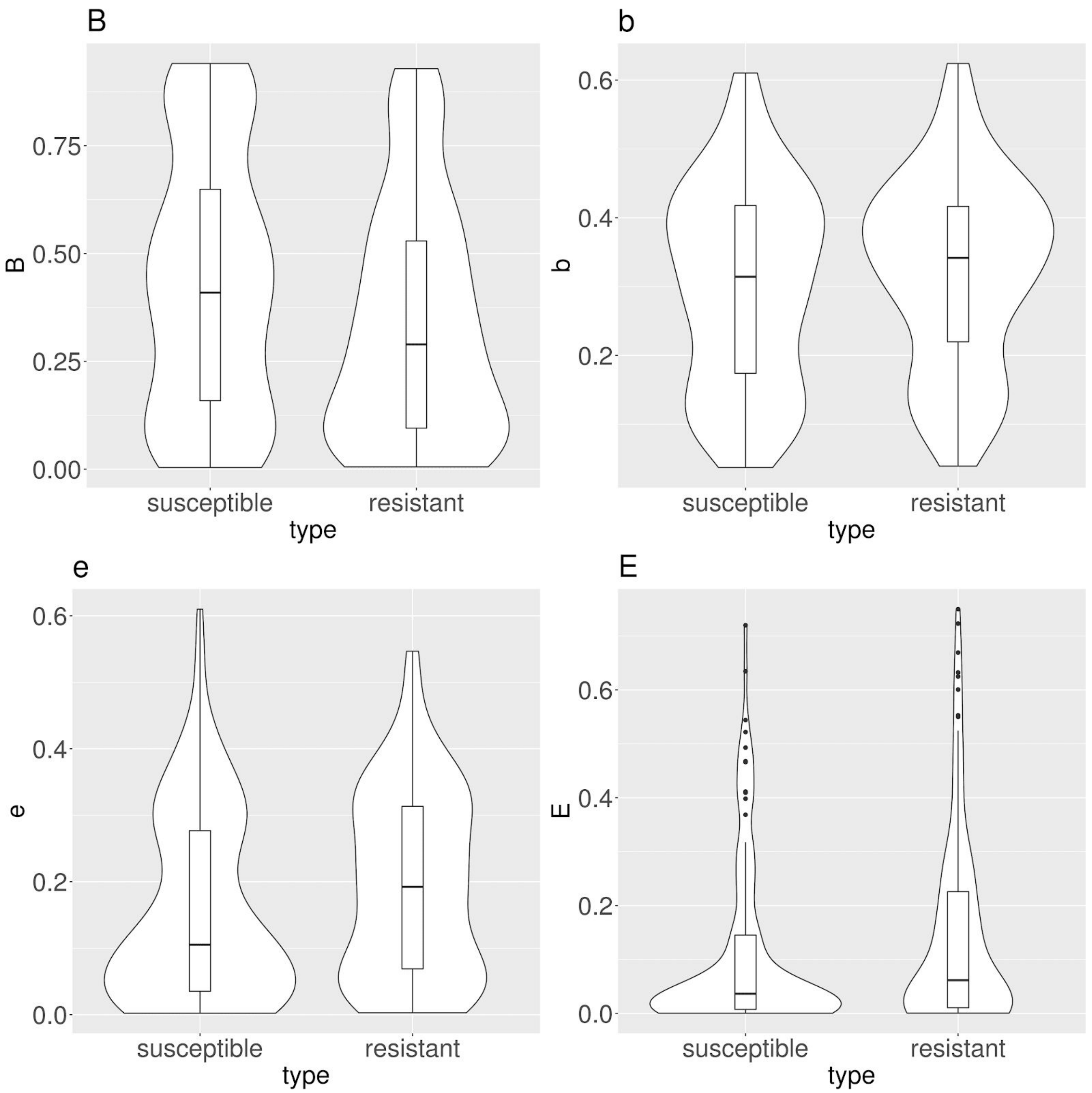

Supplementary Figure S4. Violin and box plots of the solvent accessibility scores for *Mycobacterium tuberculosis katG* mutations conferring resistance to isoniazid.

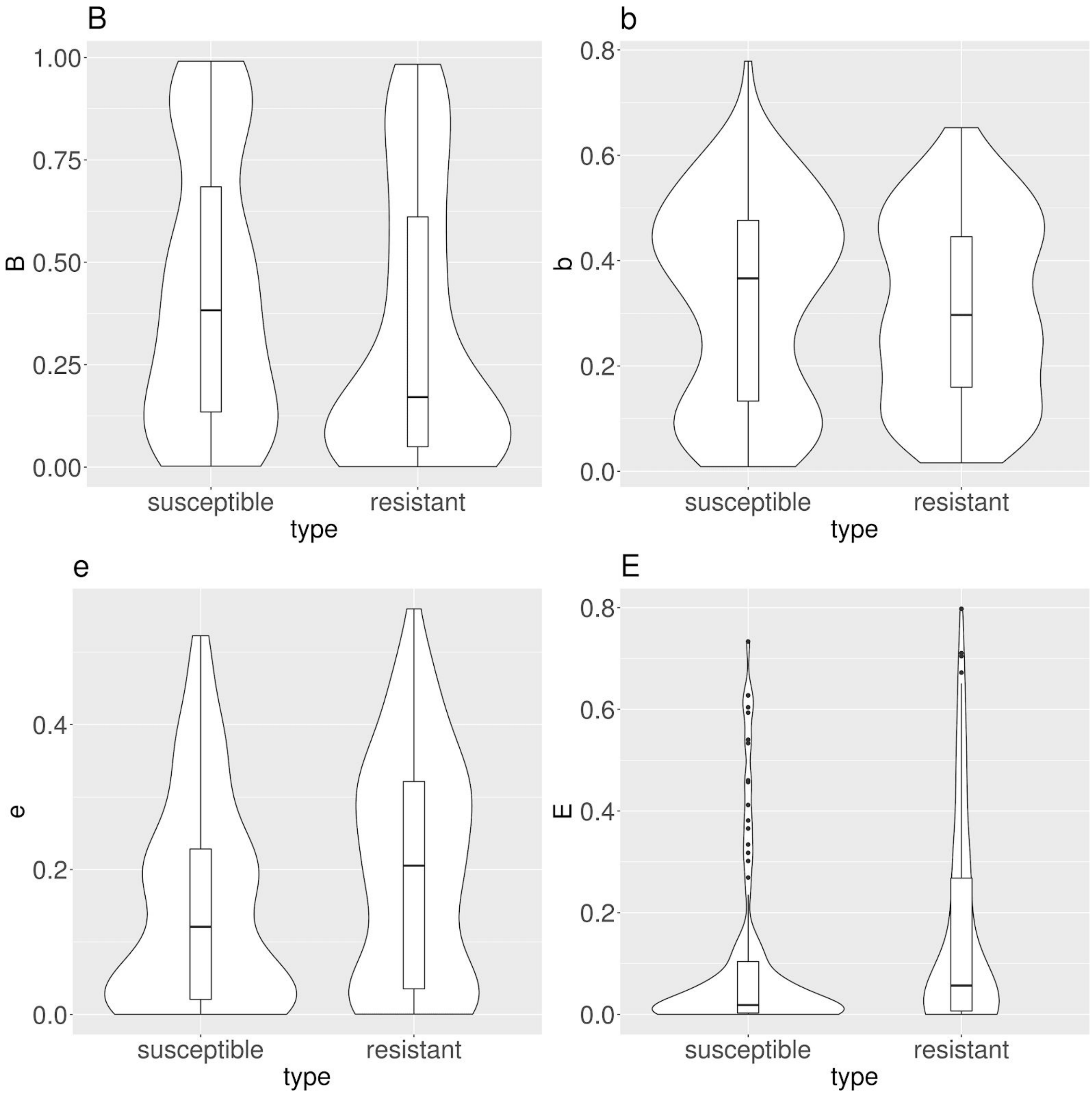

Supplementary Figure S5. Violin and box plots of the solvent accessibility scores for *Mycobacterium tuberculosis pncA* mutations conferring resistance to pyrazinamide.

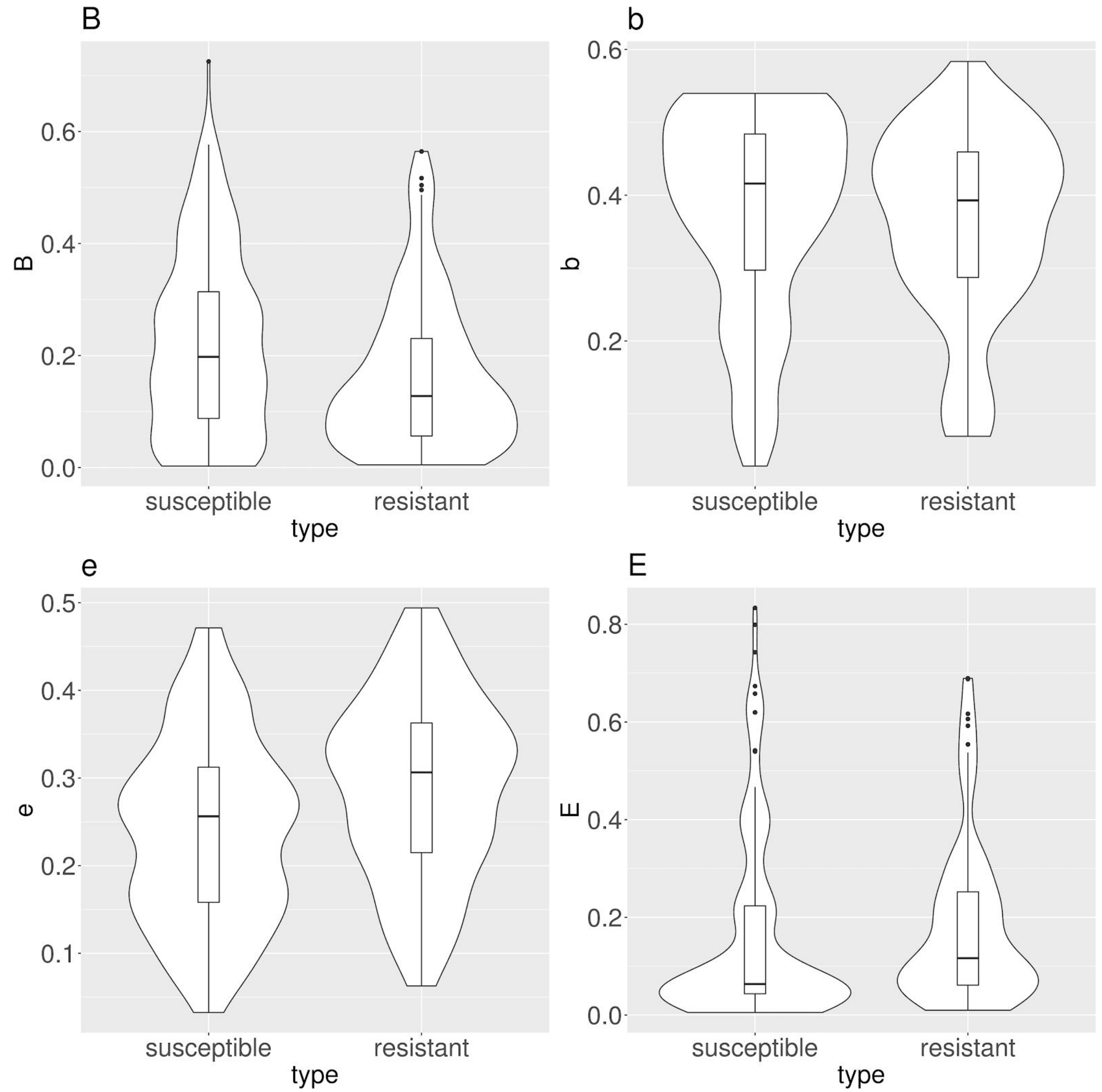

Supplementary Figure S6. Violin and box plots of the solvent accessibility scores for *Mycobacterium tuberculosis rpoB* mutants conferring resistance to rifampicin.
